# Open Molecular Crystals 2025 (OMC25) dataset and models

**DOI:** 10.1038/s41597-026-06628-2

**Published:** 2026-02-04

**Authors:** Vahe Gharakhanyan, Luis Barroso-Luque, Yi Yang, Muhammed Shuaibi, Kyle Michel, Daniel S. Levine, Misko Dzamba, Xiang Fu, Meng Gao, Xingyu Liu, Haoran Ni, Keian Noori, Brandon M. Wood, Matt Uyttendaele, Arman Boromand, C. Lawrence Zitnick, Noa Marom, Zachary W. Ulissi, Anuroop Sriram

**Affiliations:** 1 Fundamental AI Research, Meta, San Francisco, CA US; 2 Department of Materials Science and Engineering, Carnegie Mellon University, Pittsburgh, PA USA; 3 Reality Labs Research, Meta, Redmond, WA US; 4 Department of Physics, Carnegie Mellon University, Pittsburgh, PA USA; 5 Department of Chemistry, Carnegie Mellon University, Pittsburgh, PA USA

**Keywords:** Organic molecules in materials science, Cheminformatics, Computational chemistry, Density functional theory, Combinatorial libraries

## Abstract

The development of accurate and efficient machine learning models for predicting the structure and properties of molecular crystals has been hindered by the scarcity of publicly available datasets with property labels. To address this challenge, we introduce the Open Molecular Crystals 2025 (OMC25) dataset, a collection of over 27 million molecular crystal structures containing 12 elements and up to 300 atoms in the unit cell. The dataset was created by relaxing over 230,000 randomly constructed molecular crystal structures—representing approximately 50,000 organic molecules—using dispersion-inclusive density functional theory (DFT) with the Perdew–Burke–Ernzerhof (PBE) exchange-correlation functional combined with Grimme’s D3 dispersion correction (PBE+D3). OMC25 comprises diverse chemical compounds capable of forming different intermolecular interactions and a wide range of crystal packing motifs. We provide information on the dataset’s construction, composition, and properties. To demonstrate the quality and use cases of OMC25, we trained and evaluated state-of-the-art open-source machine learning interatomic potentials. By making this dataset publicly available, we aim to accelerate the development of accurate and efficient machine learning models for molecular crystals.

## Background & Summary

Molecular crystals are a class of materials characterized by the orderly arrangement of molecules in a crystalline lattice. These materials have important applications in pharmaceuticals^1–5^, organic electronics^6–9^, and other fields^10–15^, due to their unique structural and functional properties. A key phenomenon in molecular crystals is polymorphism, where a single molecule can form multiple crystal structures, influencing the physical properties of the material^16,17^. Understanding, predicting, and controlling the formation of different polymorphs is crucial for optimizing the properties of molecular crystals for tailored applications^16^. This requires exploring the potential energy surfaces of different crystal structures to gain insight into their relative stability.

Computer simulations have become indispensable tools in the study of molecular crystals. In recent years, the advent of machine learning (ML) has revolutionized the fields of chemistry and materials science^18–29^. Machine learning interatomic potentials (MLIPs) trained on large ab initio datasets offer a promising alternative to density functional theory (DFT) for predicting energies, forces, and related properties, achieving similar accuracy at a fraction of the computational cost. However, DFT provides a broader set of electronic-structure information that MLIPs currently cannot capture, making it uniquely valuable for certain applications. While DFT–particularly dispersion-corrected DFT methods–remains the standard for molecular crystal structure prediction (CSP), as demonstrated by benchmarks such as the CCDC blind test^30^, MLIPs are increasingly being integrated into CSP pipelines^31^, often serving as efficient pre-screening tools to rapidly evaluate large numbers of candidate structures before applying more computationally intensive DFT calculations^32,33^. The accuracy and computational demands of MLIPs typically range between those of classical force fields and DFT, depending on the model complexity and the training domain^34^. This middle ground enables MLIPs to strike an effective balance between computational efficiency and accuracy, making them well-suited for large-scale simulations and as complementary tools in CSP workflows. The efficacy of ML models, however, is contingent upon the quality and diversity of the training data. The development of novel MLIP architectures has been facilitated by the release of large, diverse, and open-source datasets tailored to molecules (OMol25^35^, QM9^36^, and others^37–41^) and inorganic materials (OMat24^42^, OC20^43^, and others^44–46^) applications. MLIPs used for molecular crystals have been trained predominantly on data for isolated molecules^47–51^. Some have been trained on small-scale^52,53^ or proprietary^54^ molecular crystal datasets. A significant gap remains in the availability of large-scale open datasets specifically designed for molecular crystal applications. This limitation hinders the advancement of MLIPs in this domain, underscoring the need for a comprehensive dataset that can provide a rich source of structural and property information for training machine learning models.

We present the Open Molecular Crystals 2025 (OMC25) dataset, a large-scale resource for training MLIPs for molecular crystals. OMC25 comprises over 27 million molecular crystal structures, containing 12 elements and up to 300 atoms in the unit cell. Each structure is labeled with total energy, atomic forces, and unit cell stress values. The structures comprising OMC25 were extracted from the dispersion-inclusive DFT relaxation trajectories of over 230 thousand putative molecular crystals constructed from 50 thousand unique molecules from the OE62 dataset^55^. The DFT data was acquired using the Perdew-Burke-Ernzerhof (PBE)^56^ exchange-correlation functional combined with the Grimme D3^57^ dispersion correction (PBE-D3) with tight convergence settings. Diverse sampling of molecular packing arrangements across space groups with a varying number of molecular formula units per unit cell (*Z*) was achieved using the open-source crystal generation software Genarris 3.0^58^. To thoroughly sample the potential energy landscape, including regions far from equilibrium, we generated both loosely packed and densely packed structures. To demonstrate the usefulness of the OMC25 dataset, we train MLIPs and evaluate their performance on established community benchmarks.

The dataset, model checkpoints, and code to train and evaluate models are all released open source to ensure reproducibility and to allow the community to build upon and further improve our results. We provide the OMC25 dataset with a CC BY 4.0 license. By making this dataset openly available, we aim to catalyze further research and enable advancements in structure and property prediction of molecular crystals.

## Methods

As machine learning interatomic potentials (MLIPs) see increasing adoption for use in molecular crystal research, an open, comprehensive dataset specifically designed to cover molecular crystals has become an urgent need. To achieve this, we employed a multi-step process to curate, pre-process, label, and validate a diverse set of molecular crystal structures, covering various chemical compositions, crystal systems, and space groups. The resulting OMC25 dataset includes a wide range of energetics-labeled molecular crystal structures, reflecting the rich diversity of molecular crystals found in nature and synthetic materials, and is designed to be a valuable resource for advancing materials research.

### Sampling Molecules

The OE62 dataset^55^ served as our starting point for sampling molecular structures. The OE62 dataset includes molecules extracted from the Cambridge Structural Database (CSD)^59^ repository of experimentally determined molecular crystal structures. The OE62 dataset contains 61,489 molecules comprising the elements H, Li, B, C, N, O, F, Si, P, S, Cl, As, Se, Br, Te, and I, whose geometry was optimized with DFT through the FHI-aims all electron code^60–62^ with the Perdew, Becke, and Ernzerhof (PBE) functional^56^ and the Tkatchenko-Scheffler (TS) dispersion correction^63^ until the residual forces on each atom were below 0.001 eV/Å. This dataset was sampled for molecular geometries. Potentially energetic molecules were systematically removed from the dataset using several criteria: (1) those that could not be parsed by RDKit (https://www.rdkit.org/)^64^ due to invalid SMILES were removed; (2) molecules identified as energetic materials in the CSD^59^ were excluded; (3) molecules with at least one carbon atom and a nitrogen-to-carbon ratio (N/C) of 3 or greater were removed; and (4) molecules containing nitro groups attached to nitrogen or more than one instance of potentially energetic bonds (O-O, N-N, O-N, or O/N-halogen) were filtered out using SMARTS-based substructure searches using RDKit^64^. These combined filters resulted in a final sampled set of approximately 50,000 unique molecules. Owing to the scarcity of distinct conformers for a given molecule in the OE62 dataset, only one molecular conformer was obtained in the vast majority of cases. This means that, within our data generation workflow, the molecular conformation could change only to the extent possible during final geometry relaxation in the crystal, as described below.

### Molecular Crystal Structure Generation

Random molecular crystal generation was performed with the Genarris 3.0 package (https://github.com/Yi5817/Genarris and https://www.noamarom.com/software/genarris)^58^. Genarris is an open-source software that generates diverse molecular crystal packing arrangements starting from the input molecular conformer and *Z* number, the number of molecules in the unit cell. For each sampled molecule from the OE62 dataset^55^, we selected two values of *Z* out of the six most frequent *Z* numbers in the CSD (*Z* ∈ [4, 2, 8, 1, 16, 6], in order of prevalence), with the probability distributions derived from the CSD^59^. Genarris automatically identifies all compatible space groups matching the point group symmetry of the input molecular conformer and *Z* number with up to one molecule in the asymmetric unit of the crystal (${Z}^{{\prime} }$ ≤ 1)^65^. For the purpose of generating a diverse set of random structures, as opposed to performing crystal structure prediction, we did not aim to exhaustively sample the configuration space of structures within a given space group. Therefore, for each compatible space group, we generated only two structures.

In order to train MLIPs that can work well in all regions of the potential energy surface (PES), we took advantage of the features of Genarris 3.0 to generate both loosely packed and close-packed structures. Genarris samples unit cell volumes from a Gaussian distribution around a target volume, which is estimated using the integrated machine learning (ML) model from PyMoVe^66^. To generate loosely-packed initial structures, we scaled the target volume by a factor of 1.25. Genarris avoids generating unphysical structures by demanding that the interatomic distances, *d*_*ij*_, between atoms *i* and *j* from different molecules are greater than a cutoff: ${d}_{ij} > {s}_{r}({r}_{i}^{\,{\rm{vdW}}}+{r}_{j}^{{\rm{vdW}}})$, where ${r}_{i/j}^{\,{\rm{vdW}}}$ are the van der Waals radii of atoms *i* and *j*, respectively, and *s*_*r*_ is a user-defined scaling factor. In the initial generation step, we used *s*_*r*_ = 0.95 to ensure adequate spacing between molecules. Then, the initial loosely packed structures were optimized with the *Rigid Press* algorithm, implemented in Genarris 3.0^58^ to achieve close packing. Rigid press freezes the molecular geometry and employs a regularized hard-sphere potential to optimize the molecular position and orientation along with the crystal lattice vectors to compress the unit cell as much as possible, while preserving the space group symmetry. For the Rigid Press optimization we applied the default values of *s*_*r*_ = 0.85 and specialized *s*_*r*_ values for hydrogen bonds, derived from the statistical analysis of crystal structures in the CSD to ensure that molecules are as close to each other as possible without overlap^65^.

### Sampling Random Molecular Crystal Structures

To maximize the diversity of the selected putative structures, we sampled both loosely packed structures from the initial generation stage and close-packed structures from the Rigid Press stage of Genarris. The maximum number of atoms in the unit cell was capped at 300. For each compound, chosen *Z* number, and Genarris stage, we selected up to two structures (where possible) whose geometry was converged within 5,000 iterations in the Rigid Press stage. Additionally, we sampled up to two structures from the initial generation stage, excluding the structure identifiers already sampled from the Rigid Press stage. This procedure leads to up to four structures for a given *Z* number. As noted above, we selected two *Z* numbers per molecule, producing a maximum of 8 putative crystal structures for each molecular conformer. In practice, only 4.7 structures on average were sampled per molecule. This approach allowed us to maximize the diversity of putative structures while minimizing redundancy, resulting in around 230,000 sampled putative crystal structures from Genarris.

### Structural Relaxations

The molecular crystal structures selected in the previous step were fully relaxed using dispersion-corrected DFT. The calculations were performed using the Vienna Ab initio Simulation Package (VASP, https://www.vasp.at/)^67–69^ with the projector augmented wave (PAW) pseudopotentials^70,71^. The PBE generalized gradient approximation (GGA)^56^ was combined with the Grimme D3 dispersion correction^57^. The atomic positions and lattice vectors were relaxed until the maximum per-atom residual forces were below 0.001 eV/Å, or the relaxation required more than 1,500 steps. The total energy convergence tolerance was set to 0.001 meV and the plane-wave energy cut-off was 520 eV. All VASP inputs were generated with the *RelaxSetGenerator* class of the atomate2 library (https://github.com/materialsproject/atomate2)^72^. A detailed description of additional VASP input flags is provided in the Supplementary Information. As a summary statistic, 65.6%, 83.5%, and 97.3% of structures were relaxed to maximum per-atom residual force values below 0.001, 0.01, and 0.05, respectively. The relaxation of molecular crystal structures sampled from Genarris involved over 300 million ionic steps and 1.5 billion electronic self-consistency cycles, demanding an estimated several hundred million core-hours of computational resources.

### Relaxation Trajectory Filtering

Regardless of the structural relaxation convergence, all trajectories were retained. Initially, we removed frames with non-negative energies, residual forces exceeding 50 eV/Å, and stresses above 80 GPa from the trajectory. Subsequently, if the final volume or the volume immediately following the first step deviated from the initial volume by more than 33%, the entire trajectory was discarded. However, if only a specific frame’s volume differed by more than 33% from the initial one, that frame alone was removed. This was done to make sure that the initially selected k-point density was still deemed sufficient for the ionic step. Rarely, we observed that in some cases molecular fragments broke apart or merged during relaxation. To detect this, the connectivity of each structure frame was represented as an undirected graph object in NetworkX (https://networkx.org/)^73^ from the *StructureGraph* object of *graphs* module obtained using the *JmolNN* class from the *local_env* module of *pymatgen.analysis* (https://pymatgen.org/)^74^ using default parameters. Different frames were compared with an exact graph isomorphism check as implemented in the *isomorphism* module of *networkx.algorithms*^73^. If the molecular connectivity after the first relaxation step differed from the initial connectivity, the entire trajectory was discarded. Otherwise, any frames with altered connectivity compared to the starting structure were removed. This was done to make sure that the structure optimization was not heading towards a non-realistic molecular crystal or leading to chemical reactions.

### Structure Sampling

For the purpose of training MLIPs, it is important to sample different regions of the potential energy landscape, both around the local minima and far from them. Therefore, after filtering the relaxation trajectories, a sampling strategy was employed to select a representative subset of frames. The goal was to capture the most informative and diverse set of structures along the relaxation trajectories while minimizing redundancy. A subset of up to 100 structure frames was sampled from each of the remaining trajectories, with the goal of maximizing their absolute energy differences from the preceding structure frame. In addition to energy difference-based sampling, approximately 20 total structure frames were uniformly sampled between the first occurrences in the trajectory of structures with maximum per-atom residual forces of 0.1, 0.01, and 0.001 eV/Å. The sampling strategy resulted in the final dataset including around 120 frames per molecular crystal relaxation trajectory, on average including around 10% of all frames in the original trajectories. This led to a total sample size of 27 million structures from the relaxation trajectories.

### Dataset Splits

The sampling strategy described above yielded the final structures included in the OMC25 dataset. To train MLIPs, we created the OMC25 training, validation, and test splits. To prevent leakage and ensure data integrity, an allocation process was implemented, wherein all frames belonging to putative structures of the same compound were assigned to a single split exclusively. A 90/5/5 random sampling strategy was adopted, where 90% of the data points were assigned to the training set (Train), 5% were assigned to the validation set (Val), and 5% to the test set (Test). The dataset sizes and compositions for each split of OMC25 are presented in Table 1. MLIPs were trained and optimized using the training and validation subsets of the dataset, with their performance subsequently assessed on a held-out test set, as well as through additional evaluation tests and metrics.

**Table 1:** Size, starting molecular crystal and molecule count of different OMC25 dataset splits.

Split	Size	Molecular crystals	Molecules	Fraction (%)
Train	24,870,226	207,271	44,403	90
Val	1,386,816	11,570	2,467	5
Test	1,358,143	11,327	2,467	5
Total	27,615,185	230,168	49,337	

## Data Records

The training and validation splits of the OMC25 dataset and related files are available for download from Hugging Face at https://huggingface.co/datasets/facebook/OMC25^75^, under the CC BY 4.0 license.

We prepared all data files in the Atomic Simulation Environment (ASE) Lightning Memory-Mapped Database (LMDB) database format. LMDB format^76^ is an efficient dataset type for large scale data storage designed around the concept of key-value pairs. Users can read and query the datasets using the ASE DB API^77^, where each entry (value) is an *ase.atoms* object that includes information on lattice parameters, atomic positions and numbers, periodic boundary conditions, total structure energy, atomic forces, and unit cell stress. Additionally, for each entry, we also provide information on the Cambridge Structural Database (CSD)^59^ reference code (csd_refcode) corresponding to the molecule from the OE62 dataset (taken directly from Ref. ^55^), the *Z* value of the unit cell (z_value), genarris_step tag of either the generation (*gener*) or the Rigid Press (*press*) stage of Genarris 3.0^58^, xtal.id unique crystal identifier among putative structures from Genarris step, and sid structure identifier consisting of the above information and also including the index of the structure frame in the filtered relaxation trajectory. We also provide detailed information on all unique initial molecular crystal structures that underwent structural relaxations in the *omc25-starting-crystals.csv* file (Table 2).

**Table 2:** Description of columns in *omc25-starting-crystals.csv* describing all starting molecular crystal structures that underwent structural relaxations.

Column name	Description
csd_refcode	CSD reference code^59^ of molecule from OE62 dataset^55^
z_value	Number of molecular formula units in the crystal unit cell
genarris_step	Sampled from generation (*gener*) or Rigid Press (*press*) step of Genarris 3.0^58^
xtal.id	Unique crystal identifier among putative structures from Genarris step
split	Structure was included in the training (*train*) or validation (*val*) split
nframes	Number of frames sampled from relaxation trajectory
mol.composition, xtal.composition	Composition of molecule and crystal, respectively
mol.natoms, xtal.natoms	Number of atoms in molecule and crystal unit cell, respectively
mol.mass, xtal.mass	Molar mass in g/mol of molecule and crystal unit cell, respectively
xtal.spacegroup	Crystal space group with SYMPREC=10^−5^ symmetry tolerance

## Technical Validation

A robust dataset for machine learning interatomic potentials (MLIPs) must comprehensively sample the relevant chemical, structural, and property spaces while maintaining high data quality. Here, we detail the diversity and validation of the OMC25 dataset, highlighting both its breadth, the rigorous controls applied, and the performance of MLIPs trained on it.

### Data Quality

**Fig. 1:**
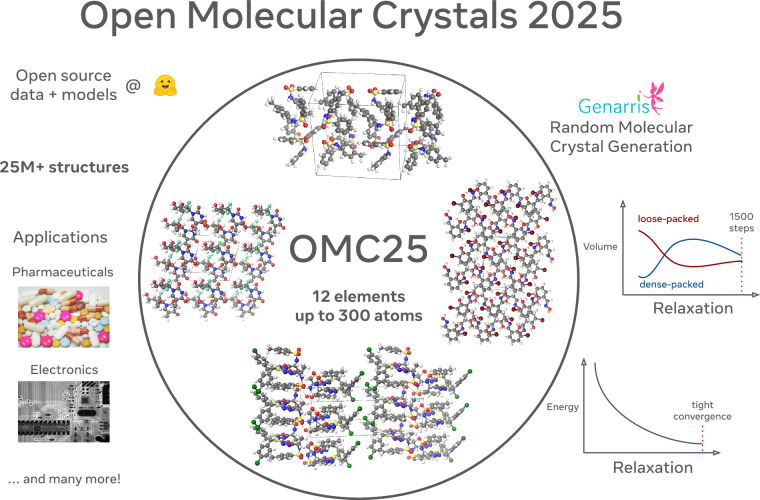
Overview of the OMC25 dataset: generation method, structure relaxations, statistics, and application areas.

The data quality and consistency were ensured through stringent design choices, including a series of filtering and sampling steps, as detailed in the Methods section. We conducted tests to ensure tight numerical convergence of the VASP settings used for the PBE-D3 calculations, as described in the Supplementary Information. Unrealistic frames were removed from relaxation trajectories by filtering based on energy, force, and stress. Consistent k-point grid density was maintained by volume filtering. Filtering based on molecular connectivity eliminated frames, in which molecular bonds were broken or unrealistically formed. The sampling strategy included frames from different stages of the relaxation trajectory, capturing configurations both far from and near equilibrium to achieve thorough sampling of the potential energy landscape. This sampling strategy provides a comprehensive representation of the system’s evolution during the relaxation process, while avoiding unnecessary redundancy in the regions near equilibrium. Notably, our dataset exhibits high consistency between the initial and final frames of the sampled trajectories. Out of 230,168 structures, only 1,516 (0.7%) had the final frame with a different space group than that of the initial frame Fig. 1.

### Chemical and Structural Diversity

**Fig. 2:**
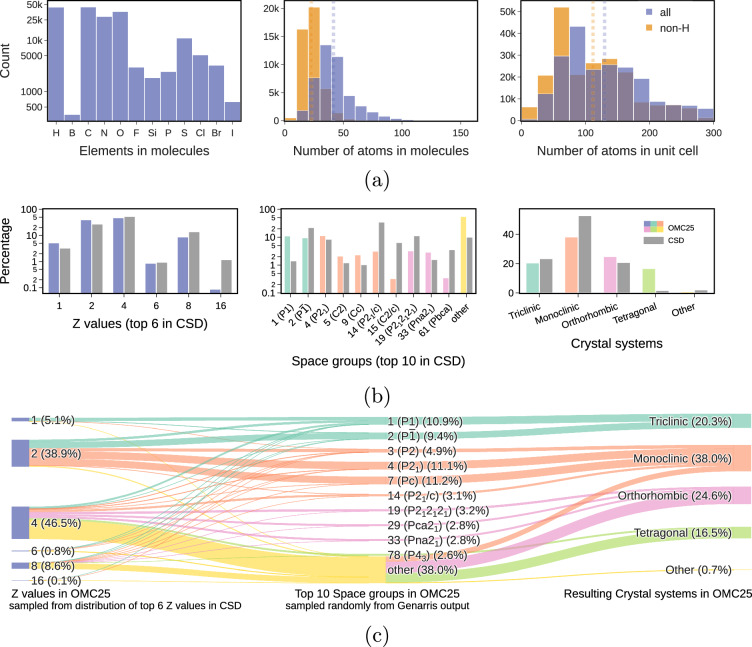
Description of the sampled molecules and molecular crystals before structure relaxations in the OMC25 training split: **(a)** elemental occurrences in molecules, distributions (histograms) and averages (vertical dotted lines) of the number of atoms with and without counting hydrogens in starting molecules and molecular crystals, **(b)** distributions of the top six most occurring Z values, the most occurring ten space groups, and four crystal systems in organic entries of CSD 6.00^59^ compared to OMC25, highlighted in yellow the collective distributions of the remaining space groups and crystal systems, **(c)** the Sankey diagram connecting *Z* values, the top ten most occurring space groups in OMC25, and resulting crystal systems, with the connecting links proportional to flow quantities. The coloring of space groups and connecting links is determined by crystal systems the space groups correspond to.

To ensure broad applicability, the OMC25 dataset was designed to capture extensive chemical and structural diversity. As shown in Fig. 2a, the OMC25 training split encompasses 12 elements most common in organic entries of the Cambridge Structural Database (CSD)^59^. Figure 2a also shows that the number of atoms in sampled molecules ranges from 4 to 164 with an average value of 42, and the number of atoms in the crystal unit cell from Genarris ranges from 12 to 300 with an average value of 130.

The sampling strategy involved selecting the top six *Z* values from the CSD, which led to a diverse set of space groups and crystal systems represented in OMC25 (Fig. 2b). In terms of structural diversity, the OMC25 dataset includes 167 distinct space groups across all seven crystal systems (computed with SYMPREC=10^−5^ symmetry tolerance). OMC25 features 22 space groups with more than 1% of structures versus only 10 in the CSD. In addition, there are notable differences in the prevalence of certain space groups in OMC25 compared to the CSD. Certain monoclinic space groups, such as *Pc* (No. 7) and *P*2 (No. 3), are significantly overrepresented in the OMC25 dataset, accounting for 11.2% and 4.9% of the starting molecular crystals, respectively. In contrast, these space groups are relatively rare in the CSD, with only 0.5% and 142 entries (out of over half a million), respectively. Similarly, the orthorhombic space group *Pcc*2 (No. 27) is represented at a frequency of 1.9% in the OMC25 dataset, compared to only 16 entries in the CSD. Furthermore, the tetragonal crystal system is significantly overrepresented in the OMC25 dataset, accounting for 16.5% of the structures, whereas it is relatively rare in the CSD, with only 1.5% representation. Conversely, the trigonal, hexagonal, and cubic systems are underrepresented in the OMC25 dataset, collectively accounting for only 0.7% of the structures, compared to 1.9% in the CSD. This difference may stem from not sampling *Z* = 3 and from the lowered symmetry of the relaxed molecular structures in the OE62 dataset, which may have prevented structure generation in space groups with many special Wyckoff positions. The relationship between the number of molecular formula units per unit cell and crystal symmetry is illustrated by the flow of sampled *Z* values into space groups and crystal systems in Fig. 2c. This provides a visual illustration of the relationships between the number of molecular formula units per unit cell and crystal symmetry.

### Property Diversity

**Fig. 3:**
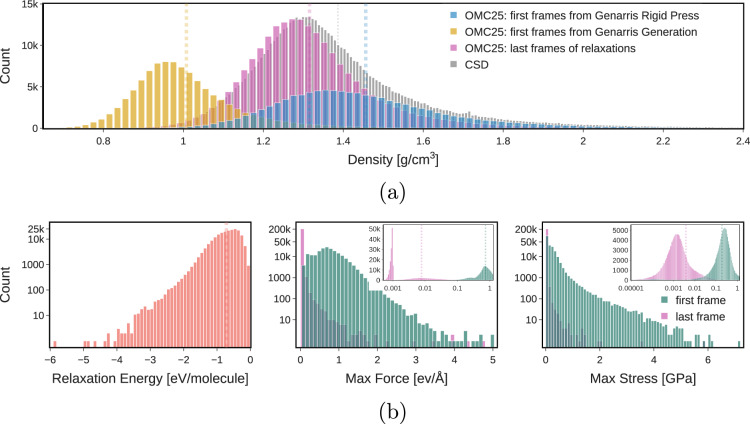
Comparison of properties from the first and last frames of relaxation trajectories of molecular crystals in the OMC25 training split: **(a)** showing density of the last frames and the first frames for different Genarris steps; overlayed is the distribution of densities of organic entries in CSD 6.00, **(b)** showing relaxation energy (the difference of energy between the initial and final frames), maximum per-atom residual force, and maximum unit cell stress. Average values are presented with vertical dotted lines.

The analysis presented in Fig. 3 reveals extensive sampling across large swaths of the potential energy landscape, which is crucial for training robust models. Figure 3a compares the density distributions of structures at various stages: initial Genarris generation, post-Rigid Press optimization, and final relaxation. The final relaxed structures closely match the density distribution of organic CSD entries, indicating realistic packing. As expected, the structures sampled after initial generation have the lowest density. Rigid Press effectively compresses the structures to achieve close-packing and significantly increases the density. The final relaxed structures, that closely follow CSD density distribution, are on average more dense than the as-generated structures but less dense than the structures compressed by Rigid Press. We sampled structures from both Genarris steps, ensuring that the final dataset includes almost equal number of both loosely and densely packed structures (Fig. 3a). Starting from either loose or dense initial structures leads to different evolution of relaxation trajectories, depending on the interplay between the intensities of intramolecular and intermolecular forces. Figure 3b presents the distributions of the relaxation energy, maximum per-atom residual force, and maximum stress in the first and last frames of the relaxation trajectories that comprise the OMC25 dataset. The relaxation energy distribution highlights the extent of structural optimization, with an average difference of 0.7 eV/molecule between the initial and final structures. The force and stress distributions demonstrate that the initial structures are far from equilibrium, while the final structures show tight convergence with the VASP settings chosen, indicating mostly finished successful relaxations. These trends are consistent across training, validation, and test subsets, confirming comprehensive sampling of the potential energy landscape and underscoring the reliability and robustness of the OMC25 dataset. Figure S2 also displays the energetics distributions for all three data splits, further demonstrating the consistency observed across the splits in the OMC25 dataset.

### Model Performance

The primary goal of the OMC25 dataset is to enable accurate, transferable predictions of molecular crystal properties using machine learning interatomic potentials (MLIPs). To this end, we adopt a model-centric validation approach: the performance of state-of-the-art ML models trained on OMC25 serves as a direct, quantitative proxy for the dataset’s informativeness, diversity, and completeness. High accuracy on relevant tasks (e.g., energy, force, and stress prediction) indicates that the dataset captures the essential physics and chemical diversity required for robust generalization. Conversely, poor model performance may reveal gaps, biases, or insufficient sampling. This approach complements traditional statistical or chemical diversity analyses and provides practical evidence of the dataset’s utility for different applications and objectives such as crystal structure prediction (CSP).

To technically validate the OMC25 dataset, we trained and benchmarked several state-of-the-art MLIPs, including UMA^20^, eSEN^22^, and EquiformerV2^29^. These models represent the current frontier in molecular and materials modeling. Model and training parameters for eSEN and EquiformerV2 are presented in Table S2 and UMA model details can be found in Ref. ^20^. All MLIPs are message-passing graph neural networks (GNNs) that operate on atomic graphs, with nodes representing atoms and edges representing neighboring atom pairs within a cutoff distance. We evaluate both energy-conserving models, which compute forces via automatic differentiation of the predicted energy (using PyTorch autograd^78^), and direct-force models, which predict forces as an explicit output. This distinction allows us to assess the impact of model architecture on predictive accuracy.

*eSEN*^22^: We selected the eSEN energy-conserving model due to its top ranking on the Matbench Discovery leaderboard^79^ for inorganic materials and its strong performance for the Open Molecules 2025 (OMol25) dataset^35^. Given its success across both inorganic materials and molecular systems, we expect it to perform well for molecular crystals.

*Universal Model for Atoms (UMA*)^20^: UMA is a versatile model trained on diverse datasets and atomic system types, including the OMC25 dataset. Its architecture integrates the strengths of eSEN with a mixture of linear experts, enhancing adaptability and accuracy. We focus on the OMC task within UMA and evaluate energy-conserving UMA models of small and medium sizes to find an optimal balance between performance and complexity.

*EquiformerV2*^29^: This direct-force GNN model incorporates transformer-inspired attention mechanisms and currently leads the Matbench Discovery leaderboard among direct-force models. We train EquiformerV2 using both a standard 6 Å cutoff and an extended 12 Å cutoff to compare the effects of model type (direct vs. energy-conserving) and receptive field size (i.e., the chemical environment an atom interacts with) on performance.

All MLIPs were evaluated on a held-out test subset, as well as on additional external benchmarks including the X23b benchmark^80^ and the Schrödinger polymorph ranking task^32^ described below. The results of these evaluations are summarized in Table 3, with further details provided in the Supplementary Information.

**Table 3:** MLIP evaluations: validation and test metrics, as well as X23b and Schrödinger polymorph ranking evaluations. The energy, force, and stress errors are presented as mean absolute errors (MAEs) for the validation and test metrics. For the X23b evaluation, the energy represents the lattice energy of the crystal, and the mean absolute percentage error (MAPE) is reported for the molar volume. For the Schrödinger polymorph ranking evaluation, the energies are normalized to the number of molecules in the unit cell. The bolded values show the best performing models, where the evaluation followed the community standards.

Model	Number of Parameters	Conserving model	Validation			Test			X23b^80^		Schrödinger polymorph ranking^32^		
			Energy ↓ [*meV*/*atom*]	Forces ↓ [*meV*/*Å*]	Stress ↓ [*meV*/Å^3^]	Energy ↓ [*meV*/*atom*]	Forces ↓ [*meV*/*Å*]	Stress ↓ [*meV*/*Å*^3^]	Lattice Energy ↓ MAE [*kcal*/*mol*]	Volume ↓ MAPE [%]	Rel. Energy ↓ MAE [*kcal*/*mol*]	Corr. Pearson ↓	Rank Corr. Spearman ↓
UMA-S-1.1 (OMC)^20^	6M^†^	*✓*	1.05	5.18	0.95	1.03	5.04	0.93	2.21	6.01	**0.35**	**0.80**	**0.74**
UMA-M-1.1 (OMC)^20^	50M^†^	*✓*	0.86	**2.92**	0.92	0.84	**2.83**	0.90	**1.94**	5.78	0.44	0.73	0.68
eSEN-S-OMC^22^	6M	*✓*	1.06	5.58	0.96	1.05	5.39	0.94	3.38	5.58	1.04	0.76	0.72
eqV2-S-OMC (6 Å)^‡^^29^	31M	✗	**0.61**	3.89	0.11	0.70	3.87	0.11	8.87	4.02	0.39	0.78	0.74
eqV2-S-OMC (12 Å)^‡^^29^	31M	✗	0.62	3.79	**0.10**	**0.67**	3.78	**0.10**	9.09	**2.50**	0.34	0.79	0.74

^†^Reported is the number of active parameters during inference, which is lower than the total number of parameters used to train UMA models^20^.

^‡^For the X23b benchmark, the single point energies of starting molecular structures in the gas phase were taken as the reference for lattice energy calculations. For the Schrödinger polymorph ranking, the single point energies of starting crystals were taken as the reference for relative energy calculations.

*X23b benchmark*^80^: We benchmark our MLIPs using the X23b dataset, a revised version of the original X23 experimental set^81^. This dataset contains 23 small to medium-sized molecular crystals featuring a variety of intermolecular interactions such as van der Waals forces, hydrogen bonding, and mixed bonding types. The evaluation task involves predicting the unit cell volume and lattice energy of each crystal at 0 K, where lattice energy is defined as the cohesive energy of the crystal relative to the isolated (relaxed) molecule in the gas phase. Figure S3a shows the reference values alongside the predictions from our MLIPs for each system.

*Schrödinger polymorph ranking*^32^: To evaluate the MLIPs performance for ranking polymorphs, we tested the models on a recent polymorph dataset from Ref. ^32^. As noted in Ref. ^48^, it is important to recognize that our MLIPs were trained on data generated with the PBE-D3 functional^56,57^, whereas the reference polymorph energies were computed using the r^2^SCAN-D3 meta-GGA functional^57,82^, which is considered more accurate. This discrepancy introduces some inherent limitations in the benchmark. For each system, we computed energy and rank correlation metrics, which were then averaged to produce the final evaluation scores. Distributions of these relative lattice energy metrics are presented in Fig. S3b.

Our results show that the OMC25 dataset can be used to train highly accurate machine learning models, with low energy, force, and stress MAEs, and for the X23b evaluation. The trained MLIPs are also well-suited for the polymorph ranking task, even considering that the reference dataset was built at a higher level of density functional theory. It is important to note that the direct force models tend to perform poorly for relaxation tasks, and thus are not recommended for such applications.

To emphasize the critical role of crystal-specific data, we conducted a comparative evaluation of identical MLIP architectures trained on the OMC25 crystal dataset and the OMol25 molecular dataset^35^. We note that the OMol25 dataset was acquired using the *ω*B97M-V range-separated hybrid meta-GGA functional with the VV10 non-local treatment of dispersion interactions^83–85^, which is significantly more accurate than PBE-D3. In addition, the OMol25 dataset was acquired using Gaussian basis sets without pseudopotentials of light atoms, although this is expected to have a more minor effect than the accuracy of the exchange-correlation functional and dispersion method. OMol25 is intended to be a general purpose molecular dataset and therefore lacks data on large clusters of organic molecules at various separations (though it does contain solvated systems and protein-ligand pockets) as those in OMC25. The differences in the performance of models trained on OMC25 vs. OMol25 may thus be attributed to the different DFT settings, the use of periodic vs. non-periodic codes, and the distributional shift of the underlying data. Our results reveal that model performance varies depending on the evaluation task (Table S1). For the X23b lattice energies, most UMA models achieve comparable accuracy, while the X23b unit cell volumes are best predicted with the OMol task of UMA. For the Schrödinger polymorph ranking task, UMA models with the OMC task show superior performance in energy metrics. This highlights that molecular and crystal datasets offer complementary advantages. This also underscores the necessity of including crystal data to fully capture the complexities relevant to molecular crystal modeling. Additional details are provided in the Supplementary Information.

### Limitations

While the OMC25 dataset and the associated MLIPs offer significant advances, several limitations remain that warrant discussion and guide future research.

First, we limited OMC25 to single-component pristine organic molecular crystals with up to one molecule in the asymmetric unit (${Z}^{{\prime} }$≤ 1). However, other classes of molecular crystals—such as co-crystals, multi-component systems, hydrates, solvates, metal-organic frameworks, and disordered systems—are of great practical interest and involve unique intermolecular interactions that remain to be explored. Future work should also prioritize richer elemental diversity beyond that in the OE62 dataset^55^. Additionally, this study used a starting single geometry-optimized molecular conformer from the OE62 dataset (except for a handful of cases). Although DFT relaxes the atomic positions in a crystal, there is no guarantee that, in practice, the resulting structures will contain highly different molecular conformers. Incorporating multiple conformers in crystal generation will be an important step toward better modeling conformer interactions and crystal energy landscapes.

Second, the level of density functional theory (PBE-D3^56,57^) employed for structure relaxations was deemed sufficient; nonetheless, several studies indicate that higher-level approaches—such as hybrid functionals like PBE0^86^ and meta-GGA functionals like r^2^SCAN^87^—along with more advanced dispersion corrections (e.g., VV10^83^, XDM^88^, TS^63^, and MBD^89,90^)—are essential for accurately capturing crystal energetics^80,81,87,91–95^. These enhancements are particularly important for CSP tasks, where high precision is required to distinguish polymorphs that differ by only a few kJ/mol^96–101^.

Third, regarding data quality, we acknowledge that maintaining a fixed k-grid is typically sufficient for minor volume changes (less than 10%). However, when the volume changes are more substantial (for example, greater than 20—33%), the k-point density may shift considerably, which can impact the reliability of computed properties. Therefore, in these situations, it is advisable to recalculate the k-grid after structural relaxation to maintain consistent sampling^102–104^.

And lastly, a known limitation of most of the current MLIPs lies in capturing long-range interactions. While message-passing layers can extend the effective receptive field beyond the cutoff distance (depending on the maximum number of neighbors allowed in the graph neural network (GNN)), accurately modeling very long-range effects extending far beyond the cutoff radius—such as those in organic-organic interfaces—remains challenging. Future work could address this limitation by incorporating recent advances such as Latent Ewald Summation (LES)^105^, which enables efficient treatment of long-range electrostatics in MLIPs. Additionally, the test and validation sets used in this study closely resemble the training data, limiting the ability to fully assess the generalizability of the MLIPs. To more rigorously evaluate these models, future benchmarks should incorporate both in-distribution and out-of-distribution test sets^106^ to better measure their transferability and robustness.

In summary, the OMC25 dataset is a unique resource, providing high-quality energetics labels for a rich and diverse set of molecular crystal structures. The MLIPs trained on OMC25 demonstrate impressive accuracy in predicting molecular crystal structures and relative energies^31^. Together, they pave the way for accelerated simulations and more reliable molecular crystal structure and property predictions, opening new frontiers in molecular crystal research.

## Supplementary information

Supplementary Information

## Data Availability

The training and validation splits of the OMC25 dataset and related files are available for download from Hugging Face at https://huggingface.co/datasets/facebook/OMC25.

## Code availability

The random molecular crystal structure generation was performed using Genarris 3.0 package^58^, which is available at https://github.com/Yi5817/Genarris and https://www.noamarom.com/software/genarris under the BSD 3-Clause license. All density functional theory (DFT) calculations were carried out with the Vienna Ab initio Simulation Package (VASP, https://www.vasp.at/)^67– 69^. The scripts to generate VASP input files with OMC25 settings are publicly available at https://github.com/facebookresearch/fairchem/tree/main/src/fairchem/data/omc. The machine learning interatomic potentials trained on the OMC25 dataset are accessible from Hugging Face: eSEN model^22^ at https://huggingface.co/facebook/OMC25 and UMA models^20^ at https://huggingface.co/facebook/UMA. The code containing all necessary information for reproducing our training and evaluation results is publicly available at https://github.com/facebookresearch/fairchem. We encourage users to cite this paper when using the dataset or pretrained models for molecular crystals in their research.
