# Supplementary material for: Open Molecular Crystals 2025 (OMC25) dataset and models
Source: Sci Data. 2026 Feb 4;13:354. doi: 10.1038/s41597-026-06628-2 (PMC12979844; doi:10.1038/s41597-026-06628-2)
Supplement: Supplementary file 1 — Supplementary Information [file 41597_2026_6628_MOESM1_ESM.pdf]

Supplementary Information:
Open Molecular Crystals 2025 (OMC25) dataset and models

Vahe Gharakhanyan[1], **Luis Barroso-Luque**[1], **Yi Yang**[2], **Muhammed Shuaibi**[1], **Kyle Michel**[1], **Daniel S. Levine**[1], **Misko Dzamba**[1], **Xiang Fu**[1], **Meng Gao**[1], **Xingyu Liu**[3], **Haoran Ni**[2], **Keian Noori**[3], **Brandon M. Wood**[1], **Matt Uyttendaele**[1], **Arman Boromand**[3], **C. Lawrence Zitnick**[1], **Noa Marom**[2,4,5], **Zachary W. Ulissi**[1], **Anuroop Sriram**[1]

[1]Fundamental AI Research at Meta, [2]Department of Materials Science and Engineering, Carnegie Mellon University, Pittsburgh, PA, USA, [3]Reality Labs Research at Meta, [4]Department of Physics, Carnegie Mellon University, Pittsburgh, PA, USA, [5]Department of Chemistry, Carnegie Mellon University, Pittsburgh, PA, USA

Correspondence: V.G. (vaheg@meta.com), A.S. (anuroops@meta.com), Z.W.U. (zulissi@meta.com), N.M. (nmarom@andrew.cmu.edu)

Contents

S1 Genarris Details **1**

S2 DFT Details **2**

S3 DFT Convergence **3**

S4 Property Distributions in OMC25 Splits **3**

S5 Evaluation Details **4**

S6 Comparison of OMC25 and OMol25 models **4**

S7 Model Hyperparameters **6**

S1 Genarris Details

```
[master]
name = <system CSD reference code>
molecule_path = <path to molecular geometry file>
Z = <sampled Z number>
log_level = debug
restart = True

[generation]
num_structures_per_spg = 2
specific_radius_proportion = 0.95
natural_cutoff_mult = 1.5
tol = 0.01
spg_distribution_type = standard
max_attempts_per_spg = 1000000
unit_cell_volume_mean = predict
volume_mult = 1.25
max_attempts_per_volume = 100000
generation_type = crystal
```

```
[symm_rigid_press]
sr = 0.85
natural_cutoff_mult = 1.2
int_scale = 0.1
method = BFGS
tol = 1e-3
maxiter = 5000
debug_flag = True
```

Listing S1 The base Genarris 3.0 [1] configuration file used for random molecular crystal generation.

S2 DFT Details

VASP version 6.3 [2–4] was used for all calculations. Calculations were executed across various machine sizes and processor types using Elastic Compute within Meta's private cloud [5], with parallelization parameters (such as NCORE and the number of MPI ranks) adjusted to the architecture of each server. Since these servers running on Elastic Compute can be preempted at any time, a single VASP calculation could be stopped and restarted many times before convergence criteria had been satisfied. With each restart, the POSCAR file was replaced with the CONTCAR file that was present when VASP stopped last and, as WAVECAR files were not written during these calculations, wavefunctions were re-initialized each time VASP was started. VASP inputs were generated using RelaxSetGenerator class from atomate2 [6]. For all structures, VASP 5.4 PBE pseudopotentials were selected, as they are suitable for the non-exotic elements present in this dataset. The atomic positions and lattice vectors were relaxed until the maximum per-atom residual forces fell below 0.001 eV/Å, or the relaxation process exceeded 1,500 steps for most crystals, although a small, randomly selected subset (around 17% of structures) was allowed to relax up to 3,000 ionic steps. The total energy convergence tolerance was set to 10^{-6} eV, and the plane-wave energy cut-off was fixed at 520 eV, based on the recommended ENCUT=1.3×ENMAX with maximum ENMAX=400 for the elements in our dataset. A maximum of 200 electronic self-consistency steps were allowed. K-point meshes were generated automatically with a default reciprocal grid density of 64, using the Γ-centered strategy. Relaxation outputs were parsed with ASE [7] and validated using several simple consistency checks.

```
ADDGRID = True
ALGO = Normal
EDIFF = 1e-06
EDIFFG = -0.001
ENAUG = 1360
ENCUT = 520
GGA = Pe
IBRION = 2
ISIF = 3
ISMEAR = 0
ISPIN = 1
IVDW = 11
LASPH = True
LMIXTAU = True
LORBIT = 11
LREAL = False
NELM = 200
NELMDL = -10
NSW = 1500
PREC = Normal
SIGMA = 0.1
```

Listing S2 Example INCAR settings for the DFT relaxations with VASP.

S3 DFT Convergence

A convergence study was conducted on 500 representative molecular crystals to validate the VASP convergence criteria for total energy. Due to computational limitations, only 484 and 73 structures were used for energy tolerance and k-point density calculations, respectively. The results are presented in Figure S1. The value used for energy tolerance (10^{-6} eV) was found to be sufficiently converged for the structural relaxations reported in this work. The final energy values showed slower convergence with respect to k-point density. The error statistics for k-point density generated by ATOMATE2 [6] was deemed sufficient for the calculations performed. This study validates that the chosen thresholds are sufficient for the calculations.

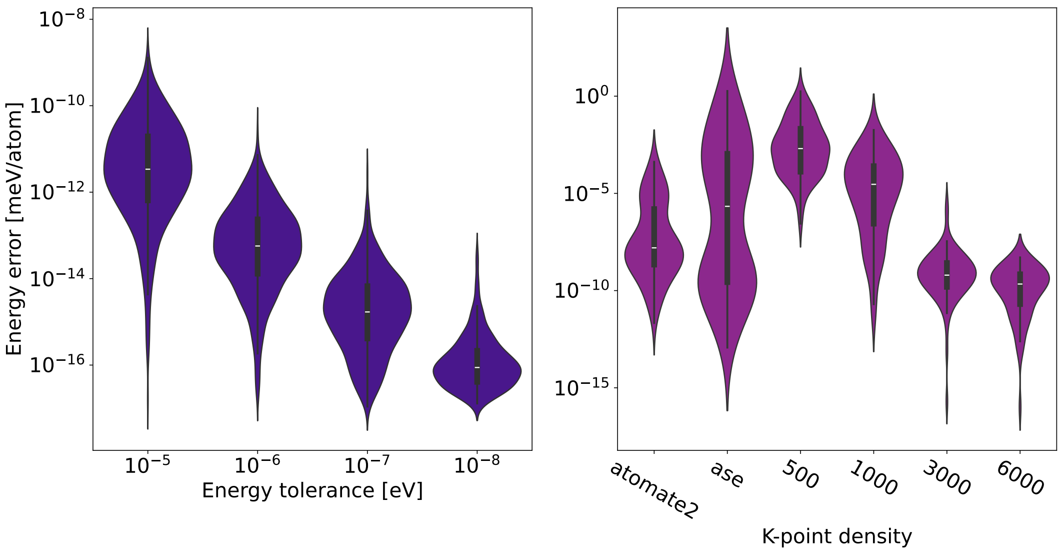

Figure S1 Convergence study for the DFT settings used in this work. We show the effect of each parameter on the energy of the structure compared to the most tightly converged settings set to 10^{-9} eV energy tolerance and 9,000 k-point density.

S4 Property Distributions in OMC25 Splits

Here, we show the energetics distributions for all three data splits, further demonstrating the consistency observed across the splits in the OMC25 dataset.

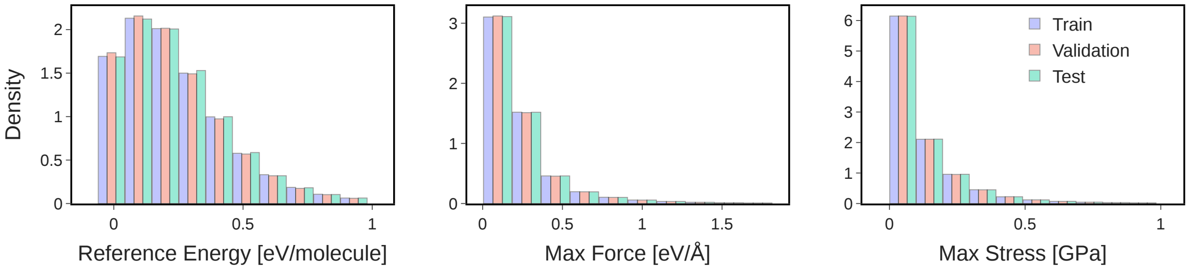

Figure S2 Distributions of energetics—energy (referenced to the last frame), force, and stress properties—for the training, validation, and test subsets, based on 10,000 randomly sampled relaxation trajectories from each split. This figure demonstrates the consistency of these property distributions across all splits. Only the 1st to 99th percentiles are displayed for clarity.

S5 Evaluation Details

X23b benchmark [8]: All starting molecular crystal structures were obtained from [8] as low temperature polymorphs and that of ammonia and carbon dioxide crystals were taken from [9] and [10], respectively. We used lattice energy reference values from [8] but the volumes were taken from [11] to remove the effects of the revised Perdew, Burke, and Ernzerhof (RPBE) [12] level of theory calculations on volumes leading to unrealistically large values for selected systems. We used ASE [7] and relaxed structures until the maximum per-atom residual forces were smaller than 0.001 eV/Å, or for a maximum of 5,000 steps, constraining the relaxation to the experimental space groups. For the direct-force models, we took the single point energies of starting molecular structures in the gas phase as the reference for lattice energy calculations.

Schrödinger polymorph ranking [13]: For each set of polymorphs of 66 systems studied, we used ASE [7] and relaxed structures with the energy-conserving models until the maximum per-atom residual forces were smaller than 0.01 eV/Å, or for a maximum of 5,000 steps. For the direct-force models, we took the single point energies. Energy and rank correlation metrics were first computed for each system (where appropriate) and then averaged to derive the final evaluation metrics. We note that OMC25 training split contains putative structures of 24 out of 66 systems included in this benchmark.

Table S1 Extended MLIP evaluation results for OMC25 and OMol25 [14] models: validation and test metrics, as well as X23b benchmark and Schrödinger polymorph ranking task. For UMA models [15], we used both the OMC and OMol tasks. The bolded values show the best performing models.

Model	Number of Parameters	Conserving model	*Validation*			*Test*			*X23b* [8]		*Schrödinger* polymorph ranking [13]				
			Energy ↓ *[meV/atom]*	Forces ↓ *[meV/Å]*	Stress ↓ *[meV/Å^3]*	Energy ↓ *[meV/atom]*	Forces ↓ *[meV/Å]*	Stress ↓ *[meV/Å^3]*	Lattice Energy *MAE [kcal/mol]* ↓	Volume *MAPE [%]* ↓	Rel. Energy *MAE [kcal/mol]* ↓	*RMSE [kcal/mol]* ↓	*Correlation Pearson* ↑	*Rank corelation Kendall* ↑	*Rank corelation Spearman* ↑
UMA-S-1.1 (OMC) [15]	6M†	✓	1.05	5.18	0.95	1.03	5.04	0.93	2.21	6.01	**0.35**	**0.43**	**0.80**	**0.60**	**0.74**
UMA-S-1.1 (OMol) [15]	6M†	✓	-	-	-	-	-	-	2.21	**2.23**	0.55	0.68	0.76	0.59	**0.74**
UMA-M-1.1 (OMC) [15]	50M†	✓	**0.86**	**2.92**	**0.92**	**0.84**	**2.83**	**0.90**	**1.94**	5.78	0.44	0.53	0.73	0.55	0.68
UMA-M-1.1 (OMol) [15]	50M†	✓	-	-	-	-	-	-	3.01	3.51	82.6 (0.61‡)	90.3 (0.76‡)	0.70	0.55	0.69
eSEN-S-OMC [16]	6M	✓	1.06	5.58	0.96	1.05	5.39	0.94	3.38	5.58	1.04	1.15	0.76	0.58	0.72
eSEN-S-OMol [14]	6M	✓	-	-	-	-	-	-	2.85	4.47	0.68	0.83	0.73	0.57	0.72

† Reported is the number of active parameters during inference, which is lower than the total number of parameters used to train UMA models [15].
‡ Reported is the result excluding five outlier systems: GLYCIN, OBEQOD, QIMKIG, QQQAUG, and UJIRIO, each with $>$ 400 kcal/mol energy error.

S6 Comparison of OMC25 and OMol25 models

To highlight the importance of crystal-specific data, we evaluated identical MLIP architectures trained on the OMC25 dataset and the molecular dataset OMol25 [14], which contains over 100 million structures with energetics computed at a higher DFT level of theory (wB97M-V/def2-TZVPD [17–19]).

The relative performance of models trained on OMC25 and OMol25 depends on the evaluation task. For the X23b benchmark [8], which involves predicting lattice energies and volumes of molecular crystals, most UMA models achieve comparable accuracy in energy predictions, with OMol25 models outperforming in volume predictions. This suggests that molecular-level data can be sufficient for predicting certain properties of crystals of small to middle-sized molecules. In contrast, for the Schrödinger polymorph ranking task [13], which requires precise energy ranking of closely related crystal structures, OMC25 models outperform OMol25 models in energy metrics, although their rank correlation scores are similar. These results underscore the critical role of explicit crystal environment data in capturing subtle intermolecular interactions and packing effects necessary for accurate polymorph evaluations. It is also important to note that the DFT levels of theory used for OMC25 and OMol25 differ from the reference level applied in the Schrödinger polymorph ranking task. When focusing on models trained solely on a single dataset (eSEN models), OMol25-trained models perform better; however, when comparing models trained on both datasets (UMA models), those with the OMC task show superior performance. Overall, the findings demonstrate the complementary strengths of molecular and

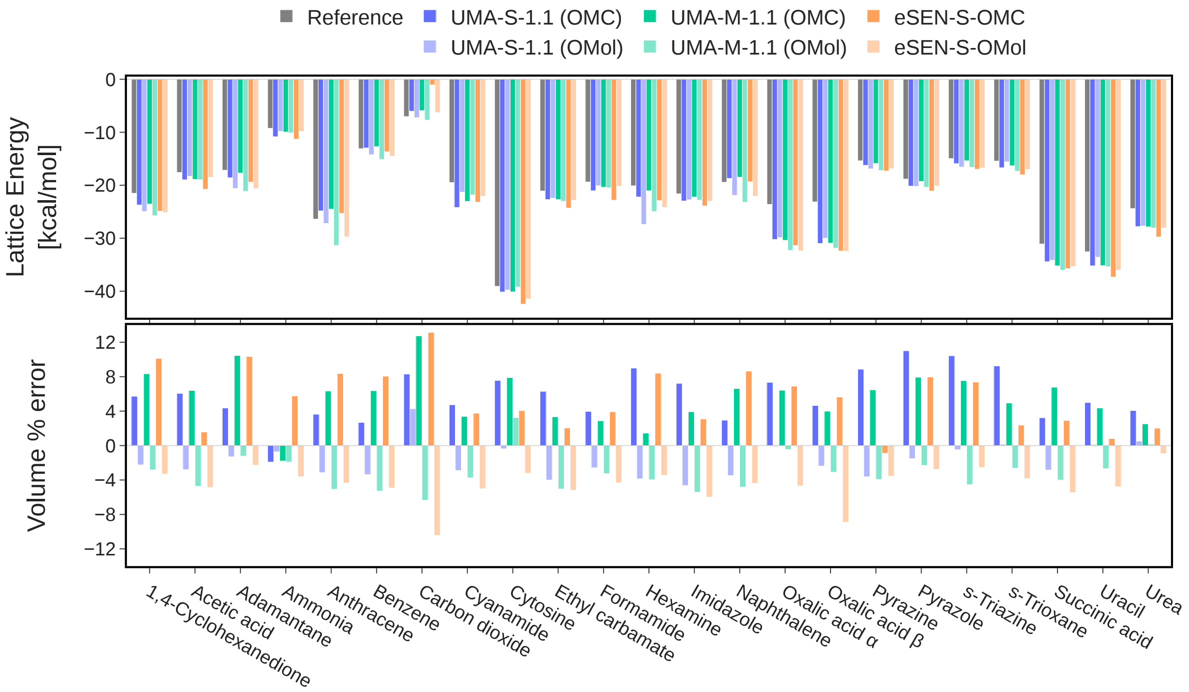

(a) X23b benchmark

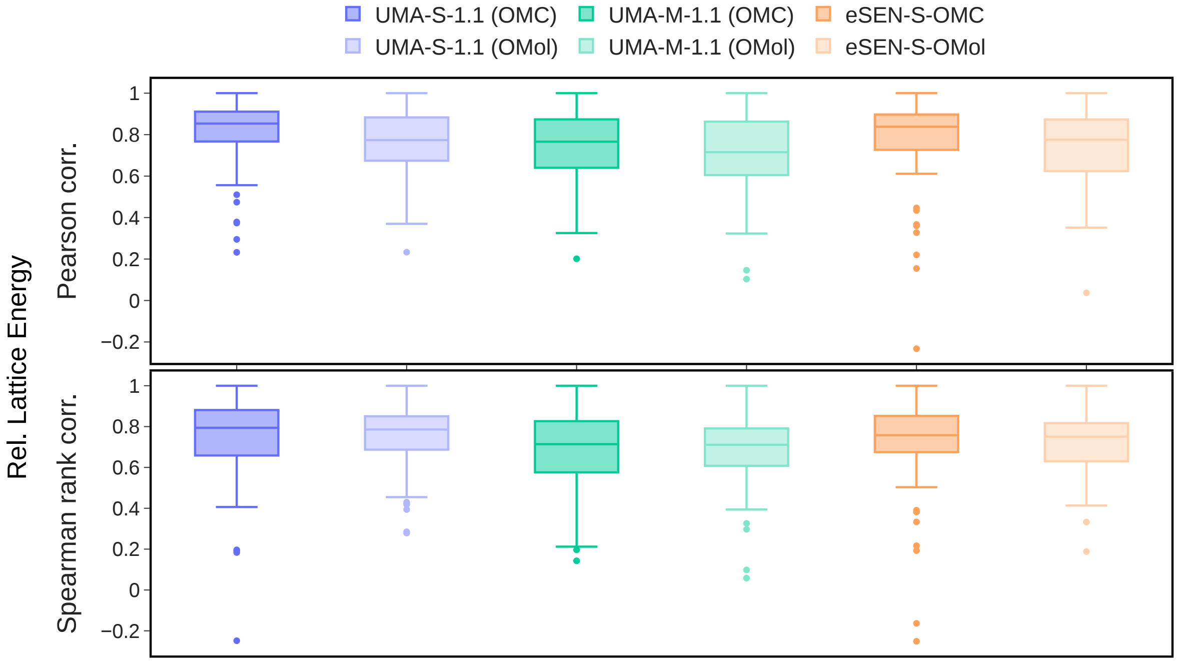

(b) Schrödinger polymorph ranking

Figure S3 MLIP evaluation results for energy-conserving models: **(a)** per system values for the lattice energies and volume percentage errors for the X23b benchmark systems, **(b)** distributions of Pearson and Spearman rank correlations of relative lattice energies for all systems considered in Schrödinger polymorph ranking.

crystal datasets depending on the specific objectives. Notably, OMC25 enables accurate modeling of molecular crystals using a dataset that is smaller by an order of magnitude compared to OMol25. This substantial

reduction in dataset size permits the use of more lightweight and efficient machine learning models, thereby increasing the accessibility of molecular crystal modeling to researchers with varying computational resources.

S7 Model Hyperparameters

Table S2 summarizes the model and training parameters for the eSEN [16] and EquiformerV2 [20] models trained on the OMC25 dataset. The eSEN model was trained in two stages: first, a direct model with a maximum of 30 neighbors and without a stress head was trained, and, subsequently, an energy-conserving model with up to 300 neighbors and an additional stress loss was trained. Detailed descriptions of the UMA models are provided in [15], and the eSEN-S-OMol model is described in [14].

Table S2 Hyperparameters and training details for the eSEN [16] and EquiformerV2 [20] models trained on the OMC25 dataset.

Hyperparameters	eSEN-S-OMC	eqV2-S-OMC
Number of parameters	6M	31M
Maximum number of neighbors	30, 300	30
Cutoff radius (Å)	6	6 & 12
Number of layers	4	8
Number of sphere channels	128	128
Number of edge channels	128	128
Maximum degree L_{max}	2	4
Maximum order M_{max}	2	2
Distance function	gaussian	gaussian
Number of distance basis	64	512
Number of hidden channels	128	-
Normalization type	rms_norm_sh	layer_norm_sh
Activation type	gate	-
ff_type	spectral	-
Number of Transformer blocks	-	8
Dimension of hidden scalar features in radial functions d_{edge}	-	(0, 128)
Embedding dimension d_{embed}	-	(4, 128)
$f_{ij}^{(L)}$ dimension d_{attn_hidden}	-	(4, 64)
Number of attention heads h	-	8
$f_{ij}^{(0)}$ dimension d_{attn_alpha}	-	(0, 64)
Value dimension d_{attn_value}	-	(4, 16)
Hidden dimension in feed forward networks d_{ffn}	-	(4, 128)
Grid resolution R	-	18
Number of GPUs	32	64
Optimizer	AdamW	AdamW
Learning rate scheduling	Cosine	Cosine
Warmup epochs	0.1	0.01
Warmup factor	0.2	0.2
Maximum learning rate	8×10^{-4}	6×10^{-4}
Minimum learning rate factor	0.01	0.01
Gradient clipping norm threshold	100	100
Model EMA decay	0.999	0.999
Weight decay	1×10^{-3}	1×10^{-3}
Dropout rate	-	0.1
Batch size	10,016 atoms	76,800 systems
Number of epochs	4, 2.4	150
Stochastic depth	-	0.1
Energy loss coefficient	10, 10	10
Force loss coefficient	30, 2	5
Stress loss coefficient	0, 1	1

References

[1] Yang, Y., Tom, R., Wui, J. A., Moussa, J. E., and Marom, N. Genarris 3.0: Generating close-packed molecular crystal structures with rigid press. *Journal of Chemical Theory and Computation* **21**, 11318–11332 (2025).

[2] Kresse, G., and Hafner, J. Ab initio molecular dynamics for liquid metals. *Physical Review B* **47**, 558 (1993).

[3] Kresse, G., and Furthmüller, J. Efficient iterative schemes for ab initio total-energy calculations using a plane-wave basis set. *Physical Review B* **54**, 11169 (1996).

[4] Kresse, G., and Joubert, D. From ultrasoft pseudopotentials to the projector augmented-wave method. *Physical Review B* **59**, 1758 (1999).

[5] Gupta, N., Narayanan, I., Handa, S., Chakraborti, S., Thapar, P., Shan, B., Rao, A., Liu, Y., Wang, P., Wu, Y., Gao, Q., Cheng, C. C.-C., You, S., Huang, L., Fan, J., Yu, K., Lin, K., Mu, T., Malani, P., Wang, H., Lu, T., and Zhang, P. Dynamic idle resource leasing to safely oversubscribe capacity at Meta. In *Proceedings of the 2024 ACM Symposium on Cloud Computing*, SoCC'24, 792–810 (2024).

[6] Ganose, A. M. *et al.* Atomate2: Modular workflows for materials science. *Digital Discovery* **4**, 1944–1973 (2025).

[7] Larsen, A. H. *et al.* The atomic simulation environment—a Python library for working with atoms. *Journal of Physics: Condensed Matter* **29**, 273002 (2017).

[8] Dolgonos, G. A., Hoja, J., and Boese, A. D. Revised values for the X23 benchmark set of molecular crystals. *Physical Chemistry Chemical Physics* **21**, 24333–24344 (2019).

[9] Hewat, A., and Riekel, C. Crystal structure of deuteroammonia between 2 and 180 K by neutron powder profile refinement. *Acta Crystallographica Section A: Foundations and Advances (Denmark)* **35**, 569–571 (1979).

[10] Simon, A., and Peters, K. Single-crystal refinement of the structure of carbon dioxide. *Acta Crystallographica Section B: Structural Science, Crystal Engineering and Materials* **36**, 2750–2751 (1980).

[11] Grimme, S., Hansen, A., Ehlert, S., and Mewes, J.-M. r2SCAN-3c: A "swiss army knife" composite electronic-structure method. *The Journal of Chemical Physics* **154**, 064103 (2021).

[12] Hammer, B., Hansen, L. B., and Nørskov, J. K. Improved adsorption energetics within density-functional theory using revised Perdew-Burke-Ernzerhof functionals. *Physical Review B* **59**, 7413 (1999).

[13] Zhou, D., Bier, I., Santra, B., Jacobson, L. D., Wu, C., Garaizar Suarez, A., Almaguer, B. R., Yu, H., Abel, R., Friesner, R. A., and Wang, L. A robust crystal structure prediction method to support small molecule drug development with large scale validation and blind study. *Nature Communications* **16**, 2210 (2025).

[14] Levine, D. S., Shuaibi, M., Spotte-Smith, E. W. C., Taylor, M. G., Hasyim, M. R., Michel, K., Batatia, I., Csányi, G., Dzamba, M., Eastman, P., Frey, N. C., Fu, X., Gharakhanyan, V., Krishnapriyan, A. S., Rackers, J. A., Raja, S., Rizvi, A., Rosen, A. S., Ulissi, Z., Vargas, S., Zitnick, C. L., Blau, S. M., and Wood, B. M. The Open Molecules 2025 (OMol25) dataset, evaluations, and models. *arXiv preprint arXiv:2505.08762* (2025).

[15] Wood, B. M., Dzamba, M., Fu, X., Gao, M., Shuaibi, M., Barroso-Luque, L., Abdelmaqsoud, K., Gharakhanyan, V., Kitchin, J. R., Levine, D. S., Michel, K., Sriram, A., Cohen, T., Das, A., Rizvi, A., Sahoo, S. J., Ulissi, Z. W., and Zitnick, C. L. UMA: A Family of Universal Models for Atoms. *arXiv preprint arXiv:2506.23971* (2025).

[16] Fu, X., Wood, B. M., Barroso-Luque, L., Levine, D. S., Gao, M., Dzamba, M., and Zitnick, C. L. Learning smooth and expressive interatomic potentials for physical property prediction. In *International Conference on Machine Learning* (2025).

[17] Mardirossian, N., and Head-Gordon, M. ωB97M-V: A combinatorially optimized, range-separated hybrid, meta-GGA density functional with VV10 nonlocal correlation. *The Journal of Chemical Physics* **144**, 214110 (2016).

[18] Hellweg, A., and Rappoport, D. Development of new auxiliary basis functions of the Karlsruhe segmented contracted basis sets including diffuse basis functions (def2-SVPD, def2-TZVPPD, and def2-QVPPD) for RI-MP2 and RI-CC calculations. *Physical Chemistry Chemical Physics* **17**, 1010–1017 (2015).

[19] Rappoport, D., and Furche, F. Property-optimized gaussian basis sets for molecular response calculations. *The Journal of Chemical Physics* **133**, 134105 (2010).

[20] Liao, Y.-L., Wood, B. M., Das, A., and Smidt, T. EquiformerV2: Improved equivariant transformer for scaling to higher-degree representations. In *International Conference on Learning Representations* (2024).
